# Advancing Teams Research: What, When, and How to Measure Team Dynamics Over Time

**DOI:** 10.3389/fpsyg.2019.01324

**Published:** 2019-06-13

**Authors:** Fabrice Delice, Moira Rousseau, Jennifer Feitosa

**Affiliations:** ^1^Brooklyn College, City University of New York, Brooklyn, NY, United States; ^2^Claremont McKenna College, Claremont, CA, United States

**Keywords:** teamwork, temporal elements, methodological tools, team phases, measurement

## Abstract

Teams are complex and dynamic entities that face constant changes to their team structures and must simultaneously work to meet and adapt to the varying situational demands of their environment (Kozlowski and Ilgen, 2006). Agencies, industries, and government institutions are currently placing greater attention to the influence on team dynamics and teamwork as they are important to key organizational outcomes. Due to increased emphasis being placed upon the understanding the maturation of team dynamics, the incorporation of efficient methodological tools to understand how teams are being measured over time becomes critical. Thus, the purpose of this paper is to present a review of relevant academic articles detailing the science behind methodological tools and general approaches to study team dynamics over time. We provide an overview of the methodological tools used to understand team dynamics with accordance to specific temporal elements. Drawing from Kozlowski et al. (1999) process model of team development, we highlight relevant emergent team constructs within each stage. As well, for each stage, we discuss the what and how to measure team dynamics. Our analyses bring to light relevant, novel and complex approaches being used by researchers to examine specific constructs within different team developmental phases (e.g., agent-based simulations, computational modeling) and the importance of transitioning from a single source methodology approach. Implications and future research are also discussed.

## Introduction

A variety of global forces have led to the continuous implementation of teams across all different areas of the modern work industry (Cross et al., 2016; O’Neill and Salas, 2018). Driven by competition and consolidation, the current workforce requires fast response time, increased levels of expertise, and shared pools of knowledge that only effective teams have the ability to bring forth (Kozlowski et al., 1999). Teams, which can be defined as “distinguishable sets of two or more people who interact, dynamically, interdependently, and adaptively toward a common and valued goal/objective” (Salas et al., 1992, p. 4), possess different attitudes, behaviors, and cognitions that are constantly shaped and influenced by that of other team members, and vice-versa (Dyer, 1984; Kozlowski and Chao, 2018). Ernst & Young Global Limited (2013) found that over 90% of organizations believe that teams increase employee participation and performance and as a result, they are adjusting accordingly to benefit the possibility of achieving these desired outcomes. For example, innovation and service-oriented organizations such as 3M and Nestlé have decentralized and instead use shared service and information centers, as well as implemented teams to maintain productivity and alignment with overall business strategies (McDowell et al., 2016).

Brought on by an influx of emphasis on teams within organizational settings, a considerable amount of research has been conducted in efforts to determine what specific characteristics actually lead to the most successful team outcomes (Humphrey and Aime, 2014). What is important to understand is that as extremely complex dynamic systems, teams consistently develop over time as members evolve and adapt to the varying situational demands they continuously face (Kozlowski and Ilgen, 2006). In addition, teams are also heavily influenced by a variety of other factors (e.g., individual personalities, working relationships amongst members of the team, roles, culture, external factors, and time) (Myers, 2013). Although researchers such as Arrow et al. (2000), have characterized teams as complex adaptive systems (CAS) and multiple theoretical frameworks have emerged to capture and explain this idea, relatively few empirical work have actually been able to examine how long it takes for teams to be effective and how these effects unfold and develop over time (Kozlowski and Bell, 2003; Ramos-Villagrasa et al., 2018; Devaraj and Jiang, 2019). In fact, most empirical studies that have incorporated the idea of emergent states within teams have mainly operationalized the various related constructs through the use of weak methodological tools, such as self-report measurements. These are often incapable of capturing temporal aspects that influence teams which only illustrate teams in a static nature (Carter et al., 2018). Therefore, though useful, self-report measures risk the creation of inaccurate conclusions, as team members may report inaccurate perceptions based on their limited ability to view all aspects of the perceived construct being measured.

Accordingly, in the past few decades, various amounts of team researchers have developed frameworks in efforts to illustrate the unpredictable course of team dynamics. However, the fact that teams are constantly and dynamically ever-changing in terms of their processes, tasks, and context makes this a very difficult task (Miller, 2003). For example, Tuckman’s (1965) theory regarding the four developmental stages of small groups (e.g., forming, storming, norming, and performing), though important to teams literature as it explains that all teams go through phases as they grow, face challenges, find solutions, and deliver results, presents limitations to team’s research because it is meant to be hierarchical in nature. In other words, teams are not able to reach the next stages unless the previous stage has been accomplished. Later developments have shown that this may not always be the case. In McGrath’s (1984) input-process-output (I-P-O) model, which has had a large influence on team dynamics research, *process* signified how members are able to combine efforts and knowledge to complete a specific task. However, despite implying team interaction, much research pertaining to process assess them only “as static retrospective perceptions” (Kozlowski and Chao, 2018, p. 578). Moreover, the I-P-O model fails to take into account that all mediational factors are not necessarily processes (Ilgen et al., 2005). Marks et al. (2001) developed a temporally based framework and taxonomy of team processes, noting that many constructs presented by researchers trying to invoke the I-P-O actually invoke emergent cognitive or affective states. Most recently, Ramos-Villagrasa et al. (2018) conducted a systematic review of the science of teams, under the logic that teams operate as CAS. As CASs, teams constantly adapt to tackle environmental occurrences, and make decisions based on the team’s history and expected outcomes of the future (Arrow et al., 2000). In examining teams through this lens, researchers are given the opportunity to view teams in a non-linear, more dynamic way. Such a method has been seen as crucial to teams research because in adapting a non-traditional lens to study teams, researchers are better able to deal with temporal issues and provide insight for better practical application (McGrath et al., 2000; Navarro et al., 2015).

All dynamic constructs are theorized to change over time, thus the use of inadequate methods of measurements often can result in inaccurate representations and unsubstantiated views of actual team dynamics. Given that no measure can ever be the perfect representation of the construct it is trying to represent and that some constructs surface and become more apparent at different stages within the team’s lifespan, researchers must consider a wider array of options to actually achieve the optimal assessment. While theories and frameworks attempt to capture team dynamics in a non-static light, not only do gaps in the literature still remain present in terms of how these dynamics can be accurately measured over time, methods of actual implementation have not progressed at a similarly. Despite being in the era of teams, teams research has not given enough consideration to temporal issues that often arise (e.g., Argote and McGrath, 1993; Kozlowski and Bell, 2003; Ilgen et al., 2005; Mohammed et al., 2009), as it is often regarded as one of the most neglected critical issue in teams research (Kozlowski and Bell, 2003). Accordingly, time should not just be regarded as the backdrop of events, but rather the lens through which the emergence of different behaviors, attitudes, and cognitions are observed (Ancona et al., 2001).

Namely, in order to effectively understand team dynamics, it is critical to examine what team emergent states and processes are most important, highlighting the *when, what*, and *how* to measure team dynamics over time. More specifically, the key challenge is to not only recognize time and temporality, but the study’s design, data collection, and the methodologies behind team dynamics (Stewart, 2010), allowing researchers to effectively replicate and understand states of team dynamics through organizational and team processes. The purpose of this current paper is to provide an overview of the methodological tools and general approaches used to understand team dynamics depending on the temporal elements. Drawing from Kozlowski et al. (1999) process model of team development in combination with an A-B-C (i.e., attitudes, behaviors, and cognitions) framework, we highlight measurement idiosyncrasies of team dynamics as the team develops. First, we conduct a systematic review of scientific articles that utilize methodological tools and general approaches to measuring team dynamics over time. Secondly, articles are coded with the intent to extract themes regarding how team dynamics are measured at team formation, task compilation, role compilation, team compilation, and team maintenance. We then provide temporal considerations in which we identify the most efficient way to capture these. Lastly, we identify opportunities to further push more rigorous research and science in terms of team dynamics measurement.

## Methodology

In these sections, we briefly summarize our theoretical and methodological approaches. Specifically, we define the scope of team dynamics and the A-B-C framework (Kozlowski et al., 1999) and describe the inclusion criteria and conceptual coding we used to inform the assumptions and their proposed revisions.

### Theoretical Approach

#### A-B-C Framework

As developing and maintaining effective teams has become a crucial topic, a myriad amount of research has been developed in an attempt to explain what conditions actually contribute to its successes and failures (Salas et al., 2015b). In an effort to consolidate key findings regarding teamwork and offer a more overarching, practical, and concise means of understanding it, Salas et al. (2008) developed the A-B-C framework for understanding teamwork. Three important aspects to teamwork that the framework depicts include the attitudes, shared behaviors, and cognitions of the individuals that make up the team. Arrow et al. (2000) define the attitudes, behaviors, and cognitions among team members as local dynamics, as they exist within the context of that specific team. Conceptually, team dynamics are embedded within team performance and are comprised of a set of these interrelated attitudes, shared behaviors, and cognitions, all of which contribute to the dynamic processes of performance. Shared behaviors specifically describe what team members do (e.g., communication, collaboration, conflict, and leadership styles). Attitudes, or what team members believe or feel include openness, trust, cohesion, and team viability. Cognitions, which include transactive memory, shared mental models, information and knowledge exchange, are what team members think or know.

These behaviors, attitudes and cognitions are in part what makes teamwork an adaptive, dynamic, and episodic process that is instrumental toward being able to achieve a common goal. The combined efforts of teamwork are necessary for effective team performance and positive outcomes, as it defines how tasks and goals are accomplished in a team context. Research has shown that if team members are not able to successfully share knowledge, trust each other, be open, and coordinate behaviors, teams have an increased likelihood if failing, even if they possess an extensive amount of task relevant knowledge (Mathieu et al., 2008). The aforementioned constructs often act as “emergent states,” which means they can become present as team members interact with one another across different performance episodes (Marks et al., 2001). The limited amount of research examining the emergent states of these constructs, likely due to logistical constraints put on researchers, complicates and restrains our understanding of their temporal nature (Salas et al., 2015a). Not all findings regarding different constructs can be generalized to all teams, especially when they are not measured over the same period of time, contexts or conditions. The A-B-C framework proves extremely useful in that it captures the elements that together shape team dynamics. In identifying these elements, researchers are able to take steps to better develop practices that can promote optimal teamwork, but only when contextual and temporal aspects are also taken into account. Research has shown that to fully understand teams, how they develop and change over time must be examined as well (Gully, 2000).

#### Temporal Frameworks

It is widely understood that teams possess a past, present and future (McGrath et al., 2000). To thoroughly understand team dynamics, it is important that researchers expand our understanding of how teams develop over time. Several temporal frameworks have been developed in an effort to address the need. As discussed by Luciano et al. (2018), different temporal frameworks should be considered when examining dynamic constructs, as different forms and varieties of time can have substantial implication for our understanding of teams. Namely, developmental theories (e.g., Tuckman and Jensen, 1977; Ford, 2014) suggest that all teams change as a function of their development over time. A frequent occurrence within developmental theories is that stages build over each other at qualitatively different stages, thus suggesting that when measured it must be taken into account that different teams may develop at dramatically different paces. Further, episodic models (e.g., McGrath, 1991; Marks et al., 2001; Jarvenpaa and Majchrzak, 2016) suggest that teams can complete different tasks within different time frames, all whilst being directed at the same goal. In other words, a common theme amongst episodic models and theories is that different processes are activated at different times based on the specific demands of the team’s tasks, implying that in order to measure dynamic constructs more accurately, they must be measured at different times as they relate to the cyclical patterns of team activity (Luciano et al., 2018). Other temporal frameworks (e.g., Barley, 1986; Weiss and Cropanzano, 1996; Park, 2010) dictate that external stimuli, such as environmental events also influence internal team processes. This implicates that research should also focus on assessing constructs before, during, and after the occurrence of such environmental events as a way to fully understand the dynamic nature of teams (Luciano et al., 2018).

### Methodological Approach

#### Literature Search and Inclusion Criteria

**FIGURE 1:**
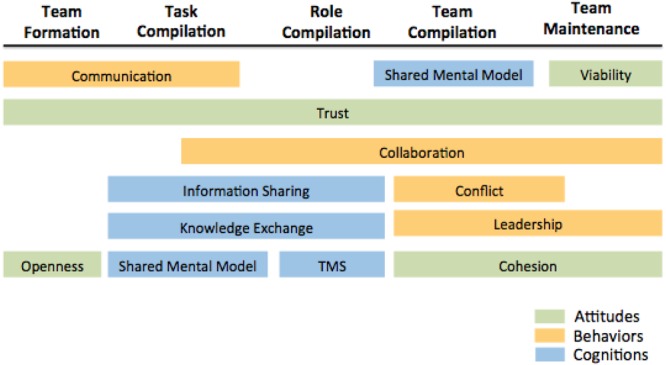
Model illustrating constructs present during team developmental stages.

This review collected and examined relevant articles that presented methodological tools and general approaches in measuring team dynamics overtime. Articles were accumulated through the use of research database sources. Searches were utilized through the electronic search engines EBSCOhost with PsycINFO and Business Source Complete being the main electronic databases. In order to generate a targeted collection of findings, we had to undergo a number of steps to find emergent processes within team development. First, we explored team emergent processes in regards to team attitudes, behavior, and cognition by examining a literature review on the role of intra-team state profiles by Shuffler et al. (2018). Second, two of the authors garnered a list of specific constructs that develop within intra-team development by examining Kozlowski and Bell (2003), who wrote an extensive review chapter on the creation, development, and operation of work teams within the different phases of team’s life cycle. As well, Taras et al. (2010) meta-analysis was also used as a reference for team emergent processes (e.g., group cohesiveness, trust, and conflict). Two of the authors held a meeting to discuss the most prominent team constructs by using the three articles to cross reference and come to a consensus. In all, four constructs across the attitude, behavior, and cognition model were developed as illustrated by Figure 1.

Our next step involved conducting a computerized search for each construct within the research database EBSCOhost. Using PsycINFO and Business Source Complete, we reviewed relevant articles through the combination of teams and the four emergent processes within the conceptual categorization of attitudinal, behavioral, and cognitive team constructs (see Table 1 for a list of the final constructs). For instance, within EBSCOhost, researchers applied transactive memory system (TMS) within the first field option and teams within the second field option. As mentioned, only relevant articles were used with each search item displaying the title, authors, keywords, and abstracts. Two authors coded 50 articles for each search item in order to extract the most significant studies as well as keep the searches consistent. In all, 600 articles were examined.

**Table 1:** Summary of literature search findings.

Constructs	Relevant articles pulled	Novel measurements
**Attitudes**		
Openness	25	
		• **Mind game lab experiment:** tested the interactions between cultural intelligence and openness on the perception of task performance (Duff et al., 2012). • **Distributed Dynamic Decision-Making Simulation:** used to examine how the performance of diverse teams is affected by member openness to experience and the extent to which team reward structure emphasizes intragroup differences (Homan et al., 2008). • **Strategic Decision-Making Simulation:** participants practiced decision-making and leadership skills in team contexts (Quigley, 2013).
Trust	28	
		• **Longitudinal experiment:** used repeated investigations of the same participants over three stages of collaboration to measure the influence of facilitated collaboration principles on trust development in global virtual collaboration (Cheng et al., 2016). • **Collaborative experiential learning approach:** tested the effects of collaborative learning on the development of cultural intelligence, trust, and global and local identity in virtual multicultural teams (Erez et al., 2013). • **High-fidelity simulation task:** participants completed a sequence of performance episodes to study the temporal variations in the buffering effect of trust in teammates (Burtscher et al., 2018).
Cohesion	28	
		• **Team laboratory experiment:** used team task involving analyzing a business case to examine the role of team political skill in predicting team effectiveness (Lvina et al., 2018). • **Comparative Performance Assessment:** used to test the antecedents and performance outcomes of social cohesion across three levels (e.g., within team cohesion, between team cohesion, and between firm cohesion (Shaner et al., 2016). • **Experiential team learning:** team members engaged in various team-based tasks and activities with their fellow teammates to understand how and in what conditions team charter quality affects team performance (Courtright et al., 2017). • **Longitudinal laboratory experiment:** used to examine the effects of intervention strategies combining team feedback and guided reflexivity on virtual teams’ affective outcomes and the mediating role of perceived social loafing in this relationship (Peñarroja et al., 2017). • **Three-wave longitudinal organizational simulation:** Participants were charged with three creativity tasks to examine the role of collective engagement in the relationship between team cohesion and team creative performance (Rodríguez-Sánchez et al., 2017). • **Dynamic Decision-Making Simulation:** Teams participated in firefighting scenarios to examine the relationships between coordination, action processes and trust and team performance (Hagemann and Kluge, 2017). • **Time series analysis:** Used temporal properties to examine the way changes in task-cohesion and shared understanding were experienced over time in sports teams (Bourbousson and Fortes-Bourbousson, 2017).
Team Viability	21	
		• **Computer game based simulation:** Examined the relationship between leadership and team viability, mediated by task cohesion through team based game that required team to run a fictional city (Curral et al., 2017). • **Computer game based simulation:** Participants performed simulated search and capture tasks to understand the relationship between team cognitive ability and personality composition (Resick et al., 2010). • **Videotape and software coding:** Developed a temporal account of team interaction by recording team meetings and coding agreement and disagreement behaviors (Lehmann-Willenbrock and Chiu, 2018).
***Behaviors***		
Collaboration	16	
		• **Concept mapping:** Examined the impact of learners’ conflict resolution on deeper learning as measured by knowledge convergence in teams (Chen et al., 2018). • **Cross-border e-business website analysis:** incorporated collaboration engineering techniques to examine how team collaboration and trust develops in globally distributed teams (Cheng et al., 2016). • **Role-play simulation:** Helped understanding of an unfamiliar and challenging situation that require cooperation and collaboration amongst teams to improve outcomes (Hayes et al., 2018). • **Synthetic task environment:** Allowed the examination of the effect of group-level information-pooling bias on collaborative incident correlation (Rajivan and Cooke, 2018)
Communication	17	
		• **Temporal distance lab experiment:** used objective speed and product quality completion tasks to examine the direct associations between temporal distance and team performance as well as the mediating role of team interaction (Espinosa et al., 2015). • **Enterprise Social Media (ESM) task:** Online discussion threads were collected with unbounded and bounded visibility to examine communication ties as conduits to critical external resources (Van Osch and Steinfield, 2018). Conflict	24	
		• **Scenario based study:** Helped studied how nationality composition (size of national diversity or number of nationalities) and context (nature of national diversity or types of nationalities) affects perceived conflict and expected performance (Ayub and Jehn, 2018). • **IMEx Business Simulation:** Used as a tool to study the consequences of relational conflicts and conflict asymmetry experienced by team members (Boroş et al., 2017). • **Critical Incident Technique:** Helped examine cultural challenges and benefits, sources of learning, and value-based differences in critical events (Brunton and Cook, 2018). • **Concept mapping:** Used as a tool to examine the impact of learners’ conflict resolution on their learning as measured by knowledge convergence (Chen et al., 2018). • **Glo-Bus business simulation:** Used as a tool to examine team performance in relations to how teams handle friendship and conflicts (Hood et al., 2017). • **Team paintball game:** Help asses coalitional aggression through a simulated coalitional combat paradigm (Pollack et al., 2018). • **Video-coding and team decision task (intra-team negotiation):** Used as a tool to measure team members power struggles through team decision task in intra-team negotiations (Van Bunderen et al., 2018).
Leadership	30	
		• **Video recorder-eye scanning:** Help observers examine the eye gazing patterns of project teams in a meeting (Gerpott et al., 2018). • **Leadership Development Simulation (LDS):** Help examine team members risk preferences, team performance, aspirational behavior, and unwarranted risk behaviors (Lanaj et al., 2018).
**Cognition**		
Transactive Memory System	40	
		• **Blog tool and statement Q-sort:** Blog tool allowed the study of virtual teams communication, coordination, and the development of TMS (Bastida et al., 2017). • **Video game:** Examine role of relational communication within the development of TMS (Kahn and Williams, 2016). • **StarJet Airways Management Simulator:** “Study role-specific versus cross-role preparation on subsequent team-level performance in a complex decision-making task” (Linton et al., 2018. p. 45). • **Audio-video recording and Hidden profile task:** Study team discussions to assess the team process through transactive retrieval and information processing (Mell et al., 2014).
Shared Mental Model	27	
		• **Traditional ICT (synchronous text-chat):** Examine team interaction and collective mindfulness behaviors (Curtis et al., 2017). • **Face-to-face or virtual (via chat):** Examine team reflections between face-to-face interactions versus virtual chats (Konradt et al., 2015). • **hboxQ-methodology (sort photographs):** Examine participants’ cognitive structures, attitudes, and perceptions (Lingard et al., 2015). • **Dynamic team task and simulated partial system failure:** Helped examine team adaptation and performance through studying a team’s shared knowledge and standardized communication with an unforeseen change (Sander et al., 2015). • **Business simulation the Global Management Challenge:** Allowed for the examination of team performance in a fictitious business through company’s’ financial indicators, shared price and ranking relative to the other teams (Santos et al., 2015). • **Computer-based Networked Fire Chief (NFC) simulation task:** Help examine team effectiveness, team mental models, and team action patterns in the scenario of extinguishing fires (Uitdewilligen et al., 2018). • **Computer-based Networked Fire Chief (NFC) simulation task:** Used to study teams in a collaborative scenario in an emergent and dynamic environment consisting of extinguishing fires (Zhou, 2018).
Information Sharing	20	
		• **Naturalistic decision-making (NDM)- Simulation-based training**: “Examine the cognitive process that is associated with failures to execute action when a decision-maker struggles to choose between equally perceived aversive outcomes” (Alison et al., 2015, p. 295). • **Employee profile configurator:** Identify characteristics of team member and place in specific clusters to examine factors affecting trust, information sharing and communication, in virtual teams (Bhat et al., 2017). • **Mechanism design-approach:** “Mechanism selects a project, recommends (privately) to each member an individual effort level, and specifies the team members’ outcome-contingent compensation.” (Blanes i Vidal and Möller, 2016, p. 171) • **NeoCITIES- Crisis simulation:** Examine how cultural composition of teams have an impact on information sharing behaviors (Endsley, 2018) • **Synthetic task environment:** Allowed the examination of the effect of group-level information-pooling bias on collaborative incident correlation (Rajivan and Cooke, 2018) • **Crisis management simulation:** Used as a tool to investigate information processing and decision-making behaviors in multidisciplinary crisis management teams’ members participating in a crisis management training (Uitdewilligen and Waller, 2018)
Knowledge Exchange	27	
		• **WhatsApp- Information and Communication Technology (ICT):** Used to study knowledge exchange and knowledge development between team members (Priyono, 2016)

Our literature analysis consisted of all source types as we did not limit our examinations to any publication dates. The selection process involved scanning the abstract and text for empirical studies as our main concern was to examine the methodological tools that team researchers are using to measure team dynamic processes. Articles were pulled if they presented sufficient information as to the approach in which teams were being studied and if team process was measured within collective team behaviors. Theoretical studies were not included as our main focus was toward empirical team studies. With a consistent and thorough inspection, 303 articles remained for analysis. Of these 303 articles, 51 were found to use novel methodology in their examination of team dynamics (see Table 1 for details).

#### Conceptual Coding and Literature Linking

Once the remaining articles were identified, two of the authors undertook the process of coding each study into an Excel sheet. Over 20 articles were coded together and discussed. The other remaining articles were then independently coded. For each search item (e.g., cohesion and teams), the excel sheet contained the articles abstracts, methodology/general approaches, the study type (e.g., laboratory/survey, field study/focus group, etc.), types of teams (e.g., virtual, managers), construct measured input, measured used, and how the team data was analyzed. Coders also examined the mediators, moderators and construct measured outputs of each article. A final verdict for each article measurement in regards to whether being a novel tool or what can be considered as new or improved techniques that allow for innovation in assessing team process dynamics (e.g., virtual experimentation) was also established. Classic methods, on the other hand, were classified as such if they were done through self-reported questionnaires, focus groups, case studies, or interviews. Although more articles fell within the realm of attitudinal and cognitive emergent states, novel measures are being mostly applied to either these cognitive emergent states or behavioral team processes.

## Role of Team Dynamics in Team Development

In the section that follows, relevant literature is compared on the basis of the most common forms of team measurements and the new approaches that are being developed by researchers to better understand team dynamics within the different phases of team development as proposed by Kozlowski et al. (1999) team development process model. In Figure 1, we illustrate the placement of the 12 constructs in the most appropriate phase for measurements in either team formation, task compilation, role compilation, team compilation, or team maintenance.

### Team Formation

Team formation, often characterized by high ambiguity and self-awareness, is known to have a great impact on performance and therefore is a critical period for modern organizations (Sorkhi and Hashemi, 2015). Moreover, during team formation, through observation and exploration, team members become more familiar with each other as they start to learn and develop within their roles. This first stage within team development can often be characterized by concerns of safety and inclusion as well as high dependency on designated leaders to provide direction during this ambiguous time (Wheelan, 2003) Similarly, members also learn the goals of their team and begin to strategize how these goals can be accomplished (Kozlowski et al., 1999; Feitosa et al., 2017). In many instances, team formation can be a difficult stage because individual differences may contribute to resistance when it comes to working together with dissimilar others to achieve these common goals. Often, individuals are attracted to similar others and therefore create distinctions between in-groups and out-groups based on perceived similarities in order to reduce ambiguity (Tajfel and Turner, 1985; Turner, 1987; Ashforth and Mael, 1989). Such behaviors have the ability to impact trust, communication, information sharing, and conflict throughout the entirety of the team’s life span (Jehn and Bezrukova, 2010).

#### Key Constructs to Measure

Considering how crucial a role perception plays within team formation, an important construct used to measure teams in this phase is openness. Costa and McCrae (1992) highlight the importance of member reactions to different ideas, actions, and values in defining openness. Individuals who exhibit high openness, especially to experience, tend to be less dogmatic and rigid in their beliefs and ideas. Instead, they are more willing to consider different opinions, are more open to new situations, and are less likely to deny conflicts compared to people who low in openness (McCrae, 1987; LePine, 2003). Moreover, openness will allow individuals to get to know each other’s strengths. These aspects of openness are very much closely related to the essence of working with new team members who more often than not, are likely to have different perspectives, attitudes, and thoughts (Cox et al., 1991; Van Knippenberg et al., 2004). Openness, though often studied at the individual-level, can have the ability to set the tone for whether or not individuals will be able to trust one another and communicate differing opinions when in the context of a team throughout the developmental stages of a team.

Relatedly, trust is often initially established through self-categorization as individuals, affected by their openness, will try to identify with other team members as a means to reduces ambiguity (Turner, 1987). Team trust refers to a party’s willingness to be vulnerable to the actions of another based on positive expectations that others will perform a certain action important to the trusting party (Mayer et al., 1995). When trust is present, team members are open to taking risk, enhancing collaboration and co-operation effectiveness (Costa, 2003). Team trust has progressively been recognized as pivotal to team processes. Although little is known about how trust develops and evolves over a team’s duration cycle (Grossman and Feitosa, 2018), evidence shows that trust is present and effects teams throughout all of the different stages of the team’s life cycle (Harrison et al., 1998, 2002). It is with time and continuous interactions, verbal and non-verbal communication, and different behavioral patterns, different personal traits will reveal themselves and become the true basis for trust among individuals (Harrison et al., 1998, 2002).

#### How Constructs Are Measured

Out of the articles pulled as a result of our literature search, the most common methodological tools during the team formation used was through the forms of self-reported survey questionnaires. For example, a study by Lu et al. (2018) on openness, using a two-wave multi-source online survey with responses from 30 teams from different multicultural organizations in China, found that reduced openness hinders a diverse team’s ability to generate innovative solutions. Especially in diverse teams, a lack of communication openness can have an impairing impact on team member information elaboration and creativity, later on. Bond-Barnard et al. (2018) conducted a self-reported survey of 151 project practitioners to assess the link between trust and collaboration. Results from the study indicated that high level of trust leads to stronger collaboration between group members. Moreover, the link between high level of trust and collaboration was more likely to predict project team success. However, being that self-report measures only provide a glimpse at static individual perception and may not even accurately reflect the behaviors of team members, the use of novel methods could prove useful in understanding the dynamic nature of teams.

With a more novel approach, Erez et al. (2013) used novel methodology in examining team trust in virtual multicultural teams with a 4-week project designed around principles of collaborative experiential learning, where trust was found to strongly moderate the project’s effect on team member cultural intelligence and global identity. Participants were put through phases to get to know each other, and prepare for the virtual team project they would be participating in. In phase one, which mimics real world team formation, participants interacted in team chat rooms where they go to know each other, introducing themselves, sharing personal information, and photos of themselves. At the end of this process, participants were then given individual feedback regarding pre-project cultural values as they related to the purpose of the study. Phase two was meant to prepare participants for the team project they would be participating in later on. Phase three was described as a post experiment wrap up, where team members received feedback on their contributions to the team’s processes. This virtual team simulation proves useful in understanding the various aspects of team formation in that it touches on the outcomes of working within diverse teams at formation, team building, and task interdependence, all of which are not only of great importance to team formation, but also to the other stages of development as well. The novelty of this study lies in the fact the researchers developed and implemented a new program for acquiring global skills regarding trust, especially for virtual multicultural teams, where such individual differences could hinder trust. In these instances, simulations can be particularly informative during the team formation stage when it comes to dealing with teams in the real-world.

#### How Constructs Are Analyzed

The examination of various articles presented an assortment of relevant team measurements that are applied during the phase of team formation. Furthermore, it is also important to take consideration of how researchers analyze items within their applied measurements. Besides the typical analysis of descriptive statistics (e.g., means and standard deviation) and correlations, the most common types of analyzing tools that were assessed between the three main constructs of team formation (i.e., openness, trust, and communication) were regression analysis, mediation, and analysis of variance (ANOVA). Researchers use regression analysis to calculate the effects of casual variables and ANOVA to determine the amount of variation in the dependent variable score within the experimental conditions (Rutherford, 2001). Moreover, mediation holds great importance as an analytical tool due to its ability to examine whether these team constructs can serve as explanatory mechanisms between team inputs and outputs (Hayes, 2012). This is extremely beneficial when understanding how teams are becoming familiar with each other during this particular phase of team formation.

### Task Compilation

Once a team has formed, individuals will begin to shift their attention toward their own individual tasks and focus on individual task mastery to develop the necessary skills required of them (Kozlowski et al., 1999). Though members will already have specialized knowledge and training in different areas, it is within this stage that team members will learn how to practice and apply their knowledge and skills within the context of the team. Moreover, in task compilation, members will seek out information and feedback from other members. Because team cognition plays a crucial role for task compilation, it is very important to understand the ways in which teams will share, exchange, and organize knowledge and how these processes occur over time (Gibson, 2001). Often characterized as a period of counter-dependency and conflict, two inevitable aspects of this stage, members can find themselves disagreeing about team goals and proper procedures. By combining their pools of knowledge and expertise, members must develop a unified understanding of how to execute the teams goals. Though conflict may arise, it is necessary for the development of trust and a more open climate, as members will be open to each other’s ideas, even if it means they might disagree with one another (Wheelan, 2003). Teams who are able to develop effective systems for information sharing and knowledge exchange have been shown to experience greater performance outcomes (Wegner, 1987).

#### Key Constructs to Measure

One of the most studied constructs within this phase is information and knowledge sharing. Knowledge distribution across teams occurs within a variety of complex paths. Therefore, the team process of knowledge sharing is an aspect that tends to hold a great importance in the progression of team performance. Knowledge sharing is defined as “team members sharing task-relevant ideas, information, and suggestions with each other” (Srivastava et al., 2006, p. 1239). Existing knowledge within teams serves as a cognitive resource to be utilized for knowledge sharing (Argote, 1999). For knowledge sharing to occur, information that is applicable to the team’s goal must be communicated with hopes of a successful collaboration between team members. In this way, communication, the act of transferring information from one place to another, among team members plays a crucial role in team functioning (Keyton et al., 2010; Beck and Keyton, 2011). Knowledge sharing also emphasizes the exchange and combination of relevant knowledge to then be applied to specific work task (Pennington, 2008). Knowledge sharing can contribute to the creation of shared mental models, which helps explain the ability of teams to cope with difficult and changing task conditions and requirements (Cannon-Bowers et al., 1993). Cannon-Bowers et al. (1993) assert that to adapt effectively, especially within the task compilation phase, team members must be able to predict what other team members are going to do and what steps are necessary to complete those tasks. Moreover, not only is it crucial for team members to engage in effective communication with each other to produce optimal outcomes, but they must also they must be able to trust that the information they provide to one another is truthful, honest and accurate. When trust is not present within the task compilation phase, teams can face a plethora of damaging effects such as lack of cooperation and resentment (McQuerrey, 2017). Understanding the emergence of constructs such as openness, communication, and trust as they relate to teams and how they are measured proves great importance to understanding team dynamics.

#### How Constructs Are Measured

The most common way knowledge and information sharing are measured are through the use of surveys and interviews (14 out of the 20 pulled). For example, in an article by Li et al. (2018), required participants to complete a survey where information sharing as measured in relation to perceived team performance outcomes. While surveys and other self-report measurements can provide useful insight to perceptions, their use also risks biases, over-exaggeration, or low response rate. However, the use of novel measurements, that often will take temporal considerations into account, may prove more useful in capturing team dynamics in a more accurate way. For example, in a cybersecurity threat detection task simulation using, Rajivan and Cooke (2018) sought to understand the effect of group-level information-pooling bias on collaborative incident correlation analysis in a synthetic task environment and revealed that participant teams were more likely to share information commonly known to the majority rather than not. However, unaided team collaboration was inefficient in finding associations between security incidents uniquely available to each member of the team. The present study helps illustrate the effectiveness of novel methodological tools in that they have the ability to present the dynamism of complex teams. Synthetic task environments are “simulation environments purposed to recreate real-world tasks and cognitive aspects of the task with the highest fidelity possible” (Rajivan and Cooke, 2018, p. 628). The researchers used information distribution processes that mimicked processes found in real-world defense environments. Important to the task compilation stage, members were assigned ownership of specific duties, but also required to discuss and correlate information related to their team task. Lingard et al. (2015) used novel methodology in their research when they employed the use of photographic q-methodology to explore shared mental models in occupational health and safety. Q-methodology has been identified as an ideal tool to study shared mental models because they reveal member cognitions, attitudes, and perceptions and reflect their subjective views of what construct or variable is being studied (Anandarajan et al., 2006). Results give important insight into the types of team shared mental models may or may not exist in and therefore how knowledge and task related activities should be examined differently for different types of teams.

From the research presented, the task compilation phase involves behavioral and attitudinal, and cognitive constructs of great importance to team functioning. Understanding the way in which team members are able to communicate information with their peers can play an integral role in predicting how members will perform, not only in this stage but also throughout the development of the team. Thus, we recommend that both behaviorally, cognitively, and attitudinally rated anchored scales, as well as simulation/lab experiments where these constructs can be assessed, are implemented to accurately depict workplace behaviors.

#### How Constructs Are Analyzed

From the collection of relevant articles within this stage, there is a salient shift in how measurements are analyzed. For instance, coding and categorization allows for the culmination of themes within interviews and text-based documents, enabling researchers to better grasp information processing; a key element within task compilation (Swanson and Holton, 2005). Partial least squares (PLS) is a preferred method over multiple regression as it does not only allow for the combination of regression and factor analysis within similar statistical procedure, but also produces a variety of reliability and validity statistics within a theoretical model (Wold, 1982; Chin and Newsted, 1999; Konradt et al., 2015). However, a key limitation with the use of PLS is that its focus is much more geared toward prediction and not theoretical fit (Akgün et al., 2012). This is not surprising as PLS is more favorable for smaller sample size (Xiang et al., 2016), and team research often struggles with sample size issues. Structural equation modeling (SEM), on the other hand, is a preferred method over regression analysis due to the fact that it allows for the investigation of two independent variables while regression does not detect interfering effects between those two independent variables. As well, SEM is useful in research that involves latent constructs or variables that cannot be directly observed (Dao et al., 2017). In order to enhance analysis, researchers would greatly benefit in using a PLS-SEM technique as it has been found to be beneficial in predication-orientated research due to its ability to strengthen explained variance and independent variables (Dao et al., 2017). Although this method is not perfect, the use of PLS in SEM undoubtedly advances research. PLS-SEM allows for more predictors to be examined as well as shortening research time frame due to the fact that only a small sample size is needed to reflect a population.

### Role Compilation

The development of a network of role exchanges, routines, and a set of roles for team members is of accordance to the information that is shared in the role compilation phase (Kozlowski and Bell, 2008). Thus, the next phase, role compilation, ensues emergent team processes of individual inputs and team-level outcomes become more focused on the overall team’s performance outcome (Kozlowski et al., 1999; Stewart et al., 2005). Some of the most dominant constructs that are measured due to its emergence within the role compilation phase fall under team cognition known as TMSs and information sharing (Ilgen et al., 2005; Pearsall et al., 2010).

#### Key Constructs to Measure

To reiterate, the role compilation phase involves the exchange, sharing and seeking out information with relations to each team member specialized capabilities, knowledge and responsibilities within a team (Pearsall et al., 2010). These role identification behaviors relate to the different constructs involving trust (i.e., a party’s willingness to be vulnerable to the actions of another based on positive expectations; Mayer et al., 1995), collaboration (i.e., shared decision making and collective responsibility amongst interdependent parties; Liedtka, 1996), information sharing (i.e., exchanging ideas amongst members; Hu et al., 2018), and knowledge exchange (i.e., transaction of information; Bullock et al., 2013). Role identification behaviors has also been shown to be a strong predictor of TMS, or who knows what (Wegner, 1987), within the role compilation phase through team discussion of each members relevant knowledge of the task (Austin, 2003; Pearsall et al., 2010). Therefore, cognitive emergent construct is a key component within the role compilation developmental phase. Hence, we further explored the approaches researchers are taking to study such cognition within teams.

#### How Constructs Are Measured

According to the literatures pulled for TMS, 38 of the 45 empirical articles measured TMS through the use of self-reported survey studies (e.g., web-based structured questionnaires). TMS has been linked to enhancing team innovation and performance (Wegner, 1987; Choi et al., 2010) and is commonly used as a mediator (i.e., the underlying mechanism that explains a relationship) (Baron and Kenny, 1986; Howell et al., 1986). For instance, an internet-based study conducted at a Finnish research organization was used to examine if TMS would mediate the relationships between task orientation and team innovation within team members (Peltokorpi and Hasu, 2016). Results illustrated how TMS mediated the relationship between task orientation and team motivation due to team members being able to explore and refine different ideas in order to update and collaborate their specialized expertise. Chiang et al. (2014), also provided a self-reported survey to a Taiwanese electrical product manufacturing company where it was TMS had a positive mediating effect on the relationship between high commitment work systems and new product performance.

Self-reported surveys are ideal for capturing perception but vary when measuring behavior as they tend to suffer from response bias and low response rates (Jones et al., 2013; Young-Hyman, 2017). Thompson (1967) found that novel and more complex tasks increases information exchange among participants when solutions are not familiar. For instance, a business stimulation was presented to individuals at an university community after being randomly assigned to a role-specific preparation team or a cross-role preparation team in order to examine the effectiveness of different types of self-preparations on subsequent team-level performance (Linton et al., 2018). Participants were primed with role-specific preparation by being randomly assigned to one of the three director titles; marketing director, operation director, and financial director. Cross-role preparation team rotated between the three roles. Results showed that role-specific preparation in teams effectively set up the preconditions for TMS, performing better on objective measures of business performance (e.g., generating profit).

There has been a steady transition into novel approaches when studying information sharing as six out of 20 articles pulled displayed some sort of novel methodology. For example, a 2-day simulation-based training exercise of an aeroplane crash over a major city was provided to a large-scale multi agency. Researchers analyzed the frequency, type, audience, and type of communication through five subject matters to examine the cognitive processes that leads to failure of executing actions of decision-making struggles during equally perceived aversive outcomes (Alison et al., 2015). Using the novel ‘hydra’ system (i.e., immersive simulated learning platform), data was collected through communication logs coordinating decisions and actions between agencies and from within by marking communication as (1) information seeking, (2) a decision, or (3) an action. Results revealed that decision making was non-time bound, involved a multiple of agencies, subordinate goals lack identification, and information sharing of communication decreased as agencies communicated from within; distracting efficient discussions and action execution. The simulation allowed researchers to examine team decision making within different points of workplace time pressure, enhancing the relevance of the data collected to accurately display real-world contextual situations. Another novel approach involved a hidden profile task presented to teams consisting of students at a Dutch University (Mell et al., 2014). Researchers found a predicted interaction effect between TMS structure and the distribution of the task information due to TMS structure being more centralized within the disparity of metaknowledge (i.e., knowledge of who knows what), allowing for more information elaboration and team performance. This study addresses the importance of fostering meta knowledge within teams as TMS knowledge decays over time; especially after group knowledge changes (Ren and Argote, 2011). From the studies presented, there is evidence of novel approaches providing in depth analysis of team shared knowledge. This is extremely beneficial during the role compilation phase as members are exchanging knowledge and roles.

It is important to realize the effectiveness of using behaviorally-anchored rating scales (BARS) to measure team effectiveness through (1) coordination, (2) cooperation, and (3) communication during role compilation, a phase where communication in regards to role exchange and developing behavioral routines is important (Kozlowski and Bell, 2003, 2008). BARS are used to measure performance dimensions in a set of incidents that represents actual behaviors which job incumbents presented in the past (Atkin and Conlon, 1978). There is a conceptual advantage of using the BARS approach as it focuses on behaviors that differentiate successful performance as well as the increase in perceived objectivity of the rater (McIntyre and Gilbert, 1994). Hence, the integration of behaviorally anchored scales can be used to set an accurate representation of behaviors as they present “less method variance, less halo, and less leniency in ratings” in replicable task duplication of real-world organizational climates which constantly deals with complex team task (Landy and Farr, 1980, p. 18).

#### How Constructs Are Analyzed

To the role compilation phase introduces the development of role exchanges ad setting of roles through the information that is shared between team members (Kozlowski and Bell, 2008). Understanding how that information is passed is important to researchers within this particular phase. Mediation and regression analysis showed to be the most common tools for analyzing within this phase to understand the relationships between constructs and how that relationship is occurring. However, researchers would benefit by switching their focus toward PLSs analysis as it has been considered to be a powerful data analytic approach in advancing the knowledge and understanding of group development (Sosik et al., 2009). It not only allows for the combination of regression and factor analysis but also mediating effects of constructs through minimal demand of sample size (Wold, 1982; Chiang et al., 2014). SEM is considered as the best method of “confirming theoretical models within a quantitative fashion” (Schumacker and Lomax, 2010, p. 7). When researchers are developing theory in exploratory research, a PLS-SEM is considered to be the preferred method (Sarstedt et al., 2014). Hence, there are many benefits for researchers in transitioning form a mediation and regression analysis as analytical tools such as PLS-SEM are able to perform such analysis within a combination saving researchers time and allowing greater advancement in the team research phenomena.

### Team Compilation

As individuals become more familiar with team member roles and each other’s specialized knowledge or abilities, the team thus enters the phase of team compilation. Team compilation involves the process of individuals of a learning, adapting, and performing their roles due to the interdependence and role distribution amongst the team (Kozlowski et al., 1999; Feitosa et al., 2017). Due to the emergence of such behaviors, relying on their behavior and cognition allows for coordination within the team to run smoothly (Pearsall et al., 2010). However, this is dependent on the success of an accurate development of role identification within the role compilation phase (Edwards et al., 2006).

#### Key Constructs to Measure

As stated, team compilation phase involves team members becoming associated with their team members and their knowledge/abilities. During this phase, the cohesion emerges as it is considered to be a relational emergent state or developing over time (Marks et al., 2001; Salas et al., 2015a). Team research focusing on the emergent process of team cohesion is important as the social integration process of team cohesion stimulates creativity, innovation, and positive team interactions (Taggar, 2002; Hülsheger et al., 2009). However, due to lack of sufficient team cognition development in the role compilation phase, team conflict becomes a major issue that hinders information processes and team member satisfaction (Bell et al., 2012). Conflict is considered to be a multidimensional construct involving task or relationship (Jehn and Mannix, 2001; Jehn and Bendersky, 2003). While relationship conflict represents the individual’s perception of the incompatibility of their teams, task conflict is the disagreement among group members at to viewpoints and ideas about their collective task decisions but with moderate levels can help teams avoid groupthink and enhance performance (Jehn, 1995; Simons and Peterson, 2000; Bell et al., 2012). Hence, we further examine the general approaches that researchers are undergoing to measure team compilation phase of the level of team adaptation through cohesion and conflict.

#### How Constructs Are Measured

After a review of the 50 most relevant studies in regards to conflict, 24 empirical articles were pulled. The most common form of measurements for conflict was self-reported surveys for 16 articles. A myriad of research studies has found strong correlation between team conflict and team performance (De Dreu and Weingart, 2003). For instance, two self-reported questionnaires were provided to United Kingdom healthcare teams and their leaders to examine how task conflict moderates the mediated relationship between professional commitment and team effectiveness in accordance to cognitive diversity (Mitchell et al., 2018). In other words, the experience of task related disagreements between members on perspective and positions showed an increase in team members effectiveness of using such knowledge. Another example of conflict being a link to the effectiveness of team output was through a survey-based study of student teams at a large university in Western Canada (O’Neill et al., 2017). Teams were examined to understand the effects of a new team-training system for postsecondary teaching and learning activities. By implementing productive conflict or teams openly discussing disagreements about task (Jehn, 1995), students with different levels of training performance would vary. Results showed that productive conflict in teams that experience full training outperformed those with partial to no-training, as productive conflict in regards to task conflict helped improve team functioning. For these reasons, self-reported surveys can be beneficial to study team perceptions.

From the 28 articles pulled in regards to team cohesion, there was a noticeable trend of researchers incorporating advancements into methodological tools to examine the cohesive nature of teams and their performance. For example, teams consisting of students at a University were provided a simulation task of the game Sim City 4 and a questionnaire on task cohesion (i.e., collective commitment and to complete a group’s task; Beal et al., 2003; Curral et al., 2017). Researchers predicted that task cohesion would lead to a positive relationship of team performance. Interestingly, when teams had perceived a maximum amounts of team cohesion, there was a decrease in team performance. The incorporation of behaviorally anchored rating scale through the use of simulation was beneficial during this study as teams are not obstructed from completing their task which thus lead to maximum cohesion, a detrimental effect that decreases performance due to the production of groupthink (Langfred, 2004). Transitioning out of single source methodologies does indeed have its perks.

Although conflict does involve the perception of team compatibility and difference of opinions, much of the research is focused on the examination of their effects on team performance (Bell et al., 2012). Agent-based simulation (ABS) or software-based simulation to mimic the behavior of interest (Kozlowski and Chao, 2018) was presented through most of the articles pulled. For instance, data was collected from business students at a large public university in the United States through a team-based business stimulation for 4-month to test a multiplex view of how friends or non-friends and intra-team conflict (task or relationship) has different effects on team performance (Hood et al., 2017). Participants participated in a 10 weekly decision rounds which they modified and acted on new strategies based on prior performance and their own competitive positions. Conflict network was measured through the respondent’s perception of the frequency of interpersonal task conflict and relationship conflict amongst the team. Results indicated that relationship conflicts among team members of friends had a negative impact on team performance compared to non-friends who had a positive impact. This article contributed to the study of performance within teams change over time as in the accordance to changes in team conflict. Boroş et al. (2017), presented a 5-day business simulation to students enrolled in a Management Integration course to explore the effects of relational conflicts and conflict asymmetry (i.e., group members holding different perception of team conflict in their group (Jehn et al., 2010). Researchers also performed computational modeling to measure the personal and direct experiences of conflict in teams as opposed to the conflict within a group. Results indicated that some team members elicit more conflict than others which affected the evolution of team dynamics and performance; even more than the high levels of conflict together (Boroş et al., 2017). These studies present evidence of ABS and computational modeling ability to provide understanding of team emergent processes per the emulation of human behavior using a virtual system.

For the most part, behaviorally anchored rating scales are a beneficial assessment tool within the team compilation phase as researchers’ study how individuals adapt and learn within a team structure in order to perform their roles, correlating with the significance of behaviorally anchored rating scales (Campbell et al., 1973; Kozlowski et al., 1999; Feitosa et al., 2017). Especially with ABSs, agent-based modeling provides advantages to conventional simulation during situations of dynamic relationships with other agents form or dissolve (Macal and North, 2006). Interestingly, the studies presented illustrated the effectiveness of novel methodological tools as they present complex systems increasing the interaction between team members, supporting Jin and Levitt (1996) view of complex team task having correlations with increasing coordination between team members. Thus, the implementation of complex and innovated novel approaches exposes researchers to real world team measures that inadequate methodological tools lack to supply within team’s research.

#### How Constructs Are Analyzed

Through the collection of articles within the team compilation phase, there were a large number of articles using mediation and regression analysis are their main analytical tool to examine the attitudinal, behavioral and cognitive constructs that are most common within this phase. Assessing how team members are building a form of interdependence between each other and associating with each other’s knowledge and abilities is determined by using these analytical tools. However, through PLSs, this job can be done simultaneously. Indeed, PLS does have its faults as it is most concerned with prediction than a test for theoretical fit and there must be careful interpretation of estimates and as they tend to increase (Akgün et al., 2012). With careful consideration, however, PLS can provide stronger “estimates of standardized regression coefficients for model paths, which can then be used to measure the relationship between latent variables” (Huang and Chen, 2018, p. 102). As researchers gear predictive research toward PLS analytical tool, the advancement of team research can greatly benefit with this practice.

### Team Maintenance

As team members have begun to fully develop a team identity, collaborative goals, and a sense of team cohesion, the process of maintaining such team behaviors becomes a critical task. Research has shown team proficiency levels decay over time; continuous behavioral success pertaining to team compilation is at risk (Feitosa et al., 2017). Therefore, team maintenance becomes a significant phase of team development. Team maintenance behavior is interpreted as “group member behavior required for maintaining the group as a working unit” (i.e., encouraging, expressing group feelings, harmonizing, gate-keeping, setting standards) (Neufeld and Haggerty, 2001, p. 37).

#### Key Constructs to Measure

Leadership is a construct that is significant with maintenance behavior as a leaders purpose is to develop expert teams, regulate activities, and help members adapt to the ever-changing environment (Kozlowski et al., 2010). Beginning at the formation stage, members often seek guidance from leaders to provide direction for the team (Wheelan, 2003). Being that teams today often exist over long periods of time, must coordinate to perform tasks, and are subject to dynamic change over time (i.e., in terms of context, task demands, and membership), team viability must also be considered within this stage of team development. Team viability refers to the “capacity for growth and sustainability required for success in future performance episodes” (Bell and Marentette, 2011, p. 276). Despite team viability being deemed an important construct for examining team maturation, this construct is understudied. As a result, construct confusion and inconsistencies in terms of how researchers have conceptualized and operationalized the construct have actually stifled its usefulness. Thus, we further expand on the emergence of leadership and team viability to present how they are being measured within relevant studies.

#### How Constructs Are Measured

A vast amount of research has shown that leadership has a high level of influence toward employee’s enthusiasm and vitality at work (Bakker et al., 2007; Atwater and Carmeli, 2009; Perry et al., 2010; Carnevale et al., 2018). Kozlowski et al. (1996a,b) stated that leaders are the prime developers of team coherence as they lead their team within a four-step learning cycle; (1) goal-setting, (2) performance monitoring, (3) error diagnosis, and (4) process feedback. From the 33 articles pulled, each study examined leadership within these four-step learning cycles through self-administered questionnaires due to it being a great indicator of perspective behaviors (Young-Hyman, 2017). For example, in a study of Ethiopian Electric Utility employees, a self-administered questionnaire was provided to examine transformational leadership behavior (i.e., leaders inspiring and intellectually stimulating their team members) on the collective efficacy of employees (Jung and Sosik, 2002; Getachew and Zhou, 2018). Results revealed that transformational leadership had a significant impact on the collective efficacy of team members as those who were high in transformational leadership behaviors were able to boost the confidence level of their followers. Due to participants expressing their sense of confidence in the team to complete extended goals because of transformational leadership, the study allows this behavior to be linked to Neufeld and Haggerty (2001) description of team maintenance behavior as a phase of expressing group feelings (Langfred, 2000; Young-Hyman, 2017). Hence, survey design would be very beneficial in studying such behaviors of a leadership.

In another article, project teams at a software firm in India were examined through how perceived time pressures affect the team process and performance on either strong or weak temporal leadership or “the degree to which team leaders schedule deadlines, synchronize team member behaviors, and allocate temporal resource” (Mohammed and Nadkarni, 2011, p. 490). Temporal leadership was assumed as a moderator of the relationship between perceived time pressure and team performance. Results showed strong temporal leadership had an indirect effect on perceived time pressure and team performance while weak temporal leadership had an indirect effect on levels of perceived time pressure and team performance (Maruping et al., 2015). These findings display a strong link to how researchers study team leadership behaviors through the four-step learning cycle. These involved perceived behaviors by team members, considering survey-based questions being an effective tool to use during this stage.

From the 21 articles pulled in our review, team viability displayed the use of three novel methodologies, while the other 18 used self-report surveys. It is understandable as team viability is based off of team members’ perception of their effectiveness based on past experiences. One novel methodology was used by Curral et al. (2017). Two hundred individuals were divided into 40 teams of five. Participants were asked to engage in a simulation experiment using a PC game SimCity 4, a city building game used in past research involving work teams. This specific version was chosen because members could act more autonomously in making decisions regarding the city they chose. Participants were then asked to complete a survey involving team viability measures, among other constructs. Results from coding game play and survey responses suggest that the mediating role of viability plays in understanding team effectiveness, especially in relation to leadership and task cohesion. Lehmann-Willenbrock and Chiu (2018) also took a novel approach in developing their multi-study longitudinal research program. Two hundred and fifty-nine employees in 43 teams participated in monthly team meetings where they discussed their workflow, problems they faced, and ways to improve as a team. These team meetings were videotaped and subsequently software coded to distinguish the difference among problem solving, off-task, and agreement behaviors. Team members were then surveyed through self-report assessments. Their methodology and findings present important implications for both teams research and practical application. Namely, this research indicates that disagreements within teams actually can enhance team learning and promote effective methods of problem solving.

Although 31 out of 33 articles pulled measured leadership through the lens of self-reported survey questionnaires or in some cases interviews, the novel measurement of simulation (i.e., agent-based simulation) does have a place in studying leadership behaviors within teams. For example, in a study of multi-team systems of United States Air Force officers, convergent (i.e., single solution) versus divergent (i.e., as many alternative solutions) risk preferences expressed during planning by the leadership was believed to affect multi-team behavior and performance (Gilhooly et al., 2007; Lanaj et al., 2018). Through the use of Leadership Development Simulation, researchers examined the risk preference, multi-team system performance, and unwarranted risk behaviors within teams. From the results, divergence of risk preferences between leadership and team’s component benefited the performance and aspirational behaviors of the multi-team system due to their ability to handle risk behaviors and task time pressure overtime. In another novel measurement study, 18 observers examined 42 zero-history teams of three who collaborated for 7 weeks at a large automotive consultant project company. The observers were examined through an eye-tracking experiment to detect leadership signals within individuals as researchers argue humans possess an automated mechanism for providing higher visual attention to emergent leaders as opposed to non-leaders (Gerpott et al., 2018). First data was collected by video recording meetings of project teams and providing teams with a peer rating questionnaire on who they thought emerged as an informal leader. Observers gazes were then measured through an eye-tracking experiment after they watched 42 brief videos of the project teams. Results indicated that observers not only gazed at emergent leaders but spent longer time periods providing their attention as opposed to non-leaders. The novelty and complexity of implementing behaviorally anchored scales for leadership, a construct revolved around behaviors of individuals influencing other team members, is undoubtedly beneficial. The two novel methodological approaches of measurement presented, agent-based simulation and eye-tracking, allowed for a broader understanding of leadership as opposed to only focusing on perception through self-reported assessments.

From the research presented, the phase of team maintenance involves a multitude of aspects as the study of behavioral and attitudinal constructs are of great importance. Understanding how group members feel about their peers and organization will have a strong prediction upon the maintenance of team behavior (Neufeld and Haggerty, 2001). Thus, both behaviorally anchored scales and self- and peer evaluation scales being implemented would allow researchers to broaden their collection of data through team perspective while accurately designing workplace behaviors. Moreover, such implementations have the ability of increasing the accuracy of research on efficient team maintenance practices through an accurate work depiction during the specific developmental stage. Thus, once researchers begin to fully accommodate complex and advance methodological approaches, then team research would notice a enhanced validity in accurately depicting organizational practice and issues.

#### How Constructs Are Analyzed

Once a team has developed a firm and stable cognitive structure of each other roles and what is needed to complete the task at hand, team maintenance is critical in order to continue behavior of a working unit. Attitudinal and behavioral constructs are thus relevant to examine within this certain phase. After assessing the most common forms of analytical tools within this particular phase, coding was one of the most widely used analytical tool by researchers as they were able to assess common themes of how teams were maintaining a cohesive working relationship in order to successfully hold group structure over time and achieve a certain team goal. Regression analysis and mediation were heavily used by researchers to test the relationship between variables and why an outcome has occurred. Although we mention path analysis in previous phases, there was a lack of usage within this phase. Henceforth, this may signal that path analysis is more widely used during research involving cognitive measure as opposed to attitudinal and behavioral constructs. Lehmann-Willenbrock and Chiu (2018) provided novel modes of analysis in that they used a statistical discourse analysis to analyze the social interactions that were recorded in their longitudinal research program. Using a multilevel, time series, explanatory models approach, researchers may be better able to capture member perceptions of team viability, as well as other constructs, crucial to team effectiveness.

## The Road Ahead

This paper summarizes the state of the science regarding team dynamics measurement allowing for a more sensitive approach to temporal components. At the present time, the most commonly used form method to examine team dynamics across a multitude of constructs and team developmental phases is through the lens of self-reported surveys. However, research has taken strides in finding new ways to obtain more efficient and descriptive results with regards to team dynamic link to team efficiency (e.g., Prochazka et al., 2018). Fortunately, the study of team emergent constructs such as team conflict, cohesion, and shared mental models are noticeably incorporating more advanced and novel methodologies within the use of complex task. From the articles pulled, it is apparent that research on team cognition constructs has seen a steady influx of novel approaches conducted under team dynamic studies. However, there is a clear gap of novelty measurement across attitudinal constructs such as trust which has been found to be important within the five stages of team development (see Figure 1).

Regarding these more novel methodologies, we highlight two that are particularly promising: ABSs and computational modeling. Specifically, these methods can address sample size issue that most teams research face. Moreover, Macal and North (2006) argue that ABSs provide an advantage in understanding the interactions of agents within dynamic relationships with other agents, as well as situations of agent relationships forming or dissolving. Computational modeling uses mathematical relationships (e.g., equations) to incorporate large numbers of process mechanisms that affect behaviors simultaneously, giving researchers an advantage of analyzing a larger scope of multilevel emergence of team dynamic processes (Kozlowski et al., 2016). However, self-reported assessments hold some advantage within research as they are able to analyze larger populations, great indicators of perspective views, as well as provide insight on team interactions. Unfortunately, they suffer from low response rates, response bias, and are obtrusive by interrupting ongoing interactions between team members (Thompson, 1967; Jones et al., 2013; Feitosa et al., 2018; Golden et al., 2018; Kozlowski and Chao, 2018). More importantly, asking participants to remember certain experiences involving attitude, behavioral, and cognitive interactions over time is detrimental to the validity and acquiring of big data (Luciano et al., 2018). Salas et al. (2018) argue that relying on more than one method of measurement can reduce single-source bias as well as reduce survey respondents’ fatigue. Hence, we call forth further team dynamic research to examine the impact factor and difference of implanting novel measures as opposed to using a single source self-reported assessment in accordance to the A-B-C framework.

Although classical methods such as self-reported survey, observations, focus groups, and interviews are commonly used by researchers, traditional measurement methods are unfortunately plagued by various challenges. What sets apart articles that followed traditional method approaches as opposed to those classified as novel approaches is the way the studies model team tasks and context. Novel studies held an advantage as to the validity and reliability of their data due to team tasks and conflict vary over time (Hinsz et al., 2009). Research has called for the consideration of dynamics and contextual features through operationalizing team environments and task in order to influence the changes of behaviors that are relevant within that workplace context (Mathieu et al., 2019). This consideration will not only allow researchers to explore emergent states of team processes but analyze emergent behaviors across varying degrees of complex research design. For instance, virtual experimentation triggers environmental events, providing more validity and reliability when assessing how team members adapt and interact within those certain situation. Such advancements in complexity of relevant research design will not only increase accuracy with measuring teams within there are different phases of team development but will strengthen the understanding of group dynamics over time.

Despite multi-method research being recommended for expanding a larger scope of team interactions and reducing data bias, it is unfortunately an expensive method and somewhat difficult to practice within organizational field studies (Kim et al., 2012). Obtained data, however, has become fairly easy as digital traces such as e-mails, smart phones, and video surveillance. They provide ongoing and unobtrusive data that can be used to adapt technology to simulate real-world complex simulations while targeting emergent team processes (Kozlowski et al., 2015; Kozlowski and Chao, 2018). Furthermore, Waber et al. (2008) discuss how team interactions sensors such as sociometric badges, a smart phone device, have been developed to accumulate data involving “bluetooth to detect people in proximity with one another, infrared to detect closer face-to-face interactions, accelerometers to assess movement, and microphones to detect vocalization” (Kozlowski and Chao, 2018, p. 581). These sociometric badges are unobtrusive, provided to large numbers of participants, and have the ability to obtain real-world data over long periods of time that can subsequently be incorporated as a source for advancing ABSs and computational modeling, avoiding multiple data collection points and ultimately minimizing the use of self-reported surveys. As well, sociometric badges are much easier to compute as they take couple of minutes to input data recorded from every hour into a spreadsheet, limiting the preparation of observation notes and coding analysis (Kim et al., 2012). This holds many opportunities for future research as laboratories that may not have access to ABS or computational modeling programs would still have the ability to capture real-world team interaction behaviors over time. Thus, we call forth future research upon the use of sociometric badges as this data collection method provides a strong positive outlook for researchers to gain knowledge upon team dynamics.

To reiterate, digital traces such as e-mails, smart phones, and video surveillance is at researchers’ disposal for unobtrusive data. Luciano et al. (2018) discuss how big data is generated through three general types of data streams: (a) behaviors, (b) words, and (c) physiological responses. Sociometric badges is a perfect example of behavior-related data streams due to its ability to measure proximity, movement, or interactions with other team members (Waber et al., 2008). When analyzing word-related data streams, Luciano et al. (2018) discuss computer-aided text analysis (CATA) and Hidden Markov Model (HMM) (Pentland, 2007). CATA allows for researchers to infer what is being said through the quantifying of word use and pattern, while HMM analyzes how things are being said in accordance to the inter-relationship speech patterns (e.g., frequency, amplitude, or amount) over time (e.g., turn-taking, interruptions, variation of speaking time). Physiological data streams, such as brain activity, can be analyzed through the use of quantitative electroencephalography (QEEG; Waldman et al., 2015). Researchers are able to examine group dynamics by placing this portable hardware with sensors on an individual scalp and record electrical activities that signify human interactions such as leader emergence, collective cognition, and team members engagement. By incorporating such innovative tools, different streams of interpersonal interactions data through teams affect, behavior, and cognition can be obtained, broadening the scope of what we understand about team dynamics and emergent team processes. Thus, we call forth for their incorporation within future teams research as a way to measure naturally occurring individual and collective processes activities.

Besides the advancement in methodological tools and approaches in measuring emergent team process across a different periods of team developmental stages, analysis tools should also be a concern. We touched upon the many advantages of using PLSs within SEM. PLS-SEM is an approach that seeks to maximize the explained variance of dependent constructs through a causal modeling technique (Hair et al., 2011). PLS-SEM is beneficial in circumstances of prediction, theory development, and research involving a limited number of participants (Wong, 2013). Although PLS-SEM analytic tool is promising and holds potential for business research, there is a noticeable gap in research as it was most noticeable within studies pertaining to cognitive constructs. Due to its predictive nature, it is recommended that future research begin implementing PLS-SEM within studies involving regression-based approaches as there is much benefits in using this SEM approach as opposed to the traditional regression and mediation analysis. Especially in the process of studying relationships between latent constructs (i.e., not directly observed but inferred from other variables), researchers are able to calculate estimates of factor scores latent variable in relations to the observed indicator variable more precisely (Hair et al., 2011). Thus, in order for team research advancement, it is imperative that researchers continue to adopt innovative novel methods in order to obtain more accurate data of emergent team process across different team developmental phases and context.

There is a need for more research to examine the effects of new methodological approaches to better cultivate team research on emergent constructs in each developmental team stage. Researchers must continue transitioning to real-time measurement that is provided through innovative technological. With the application of methodological approaches that trigger relevant workplace situations accompanied by strong analytical tools in assessing these measures, research will be gifted with new found accurate measurements that will set forth new heights for understanding teams research. Unfortunately, there is a lack of meta-analyses that focused on examining a variety of team processes across different stages of team development. As well, our research could have also benefited from a meta-analysis that also addressed team process change within different types of teams. Thus, examination of team processes can change over time and type of team through a meta-analytical approach by assessing their effect sizes is recommended in order for researchers to fully examine the strength of the relationship between type of team and temporal dynamics. Researchers would be able to perceive the relationship between team dynamic changes overtime and the type of teams these changes are more likely salient within. These future recommendations will allow the progression of team research to set forth and continually adapt to the use of emerging methodologies/approaches, obtaining and analyzing team dynamic workplace data with precision; revolutionizing methodological assessments.

## Conclusion

Within the past few decades, organizations have made a salient and ongoing shift from individual-work organized jobs to a more team-centric worked based structure (Kozlowski and Bell, 2003). Accordingly, research on how individual personalities and behaviors interact in working relationships to effect teams, roles, culture, and the organizational structure comes into play within the form of team dynamics research (Myers, 2013). In this article, we address the question of how team research is conducting empirical studies to better understand the development of teams through the lens of team dynamic constructs. Through the examination of common attitudinal, behavioral, and cognitive emergent team constructs, we explore the different methodological tools/approaches being applied by team research in accordance to the developmental stages as specified within Kozlowski et al. (1999) team developmental model.

From the myriad of articles collected, researchers are taking the necessary steps by incorporating new, improved, and innovative methodological approaches to better conceptualize relationships between team emergent constructs and team developmental stages. The present work illustrates the importance of simulation-based studies as they are beneficial in cultivating a relevant working environment due to the triggering of situational based context. These situations can be done through behaviorally anchored rating scales geared toward ABSs which allows researchers to closely examine team dynamic relations within complex systems. Although these tools are available, majority of relevant studies within the past decade are relying on traditional methodological approaches, showing signs of a reliance and comfortability to outdated methods. This article is not specifically telling future research to leave traditional methodological tools (i.e., surveys, interviews, case studies, and focus groups) behind, as these methods do have beneficial factors. For instance, there is much work to be done in advancing behaviorally anchored rating scales.

Future recommendations are addressed for incorporating multi-method measurements, specifically combining traditional methodological tools with ABSs or computational modeling in order to enhance the relevance of data obtained. As well, sociometric badges, computer-aided text analysis, HMM, and quantitative electroencephalography are also expanded on as tools to measure behavioral, word, and physiological data streams for obtaining real-world unobtrusive data. These tools are advantageous in providing a stronger source of interpersonal behaviors for advancing behaviorally anchored rating scales. Especially with a shift into incorporating PLS-SEM for predictive and theory development, future teams research will benefit with more accurate score values of latent constructs through the use of smaller sample sizes. Following our recommendation to incorporate innovative approaches such as multimethod modeling and novel methodological/analytical approaches, new found team dynamic information can surely impact teams research, opening doors for better comprehension of replicating workplace environment and accumulating more accurate measurements of team processes. Although these approaches are not perfect, the steps team research should continue to take to advance our insight of team dynamics through innovative methodological and analytical practices should not go without notice as they are establishing a new scope built around the successful outlook of future team research.

## Author Contributions

FD and MR provided substantial contributions to the conception or design of the work. JF drafted the initial outline, set-up the methodological plan, and revised the manuscript critically for important intellectual content. All authors have provided approval for publication of the content and agreed to be accountable for all aspects of the work in ensuring that questions related to the accuracy or integrity of any part of the work are appropriately investigated and resolved.

## Conflict of Interest Statement

The authors declare that the research was conducted in the absence of any commercial or financial relationships that could be construed as a potential conflict of interest.
